# Acculturation and the oral health of a nationally representative sample of Hispanic children in the United States: an analysis of 2011–2012 National Survey of Children’s Health data

**DOI:** 10.1186/s12889-019-8045-x

**Published:** 2020-01-28

**Authors:** Faizan A. Kabani, Erica L. Stockbridge, Bibi Berly Varghese, Abiah D. Loethen

**Affiliations:** 1 Caruth School of Dental Hygiene, Texas A&M University College of Dentistry, 3302 Gaston Avenue, Suite 136, Dallas, TX 75246 USA; 2 Department of Health Behavior and Health Systems; School of Public Health, University of North Texas Health Science Center, 3500 Camp Bowie Blvd, Fort Worth, TX 76107 USA; 3 Department of Advanced Health Analytics and Solutions; Magellan Health, Inc., 4800 N. Scottsdale Rd. #4400, Scottsdale, AZ 85251 USA; 4 SaferCare Texas; University of North Texas Health Science Center, 3500 Camp Bowie Blvd, Fort Worth, TX 76107 USA

**Keywords:** Oral health, Dental caries, Acculturation, Social determinants, Pediatric health, Hispanic, Latino, Children, Disparities

## Abstract

**Background:**

Oral disease is a serious public health issue, and Hispanic children in the United States (US) are more likely than children of other racial/ethnic groups to experience dental caries. Although Hispanic children are a growing segment of the US population there is limited research on the association between acculturation and oral health outcomes in this population. This study examined the associations between household acculturation and pediatric oral health in the Hispanic population using a nationally representative sample of children.

**Methods:**

Data from the 2011–2012 National Survey of Children’s Health were analyzed; analyses included Hispanic children ages 1 to 17. Household acculturation was assessed with a combination of language and parental nativity, while oral health was assessed via parents’/guardians’ reports of children’s dental caries. Logistic regression was used to examine the association between acculturation and oral health, adjusting for other demographic and social determinants of pediatric oral health. We assessed significance at the *p* < 0.05 level, and all analyses accounted for the survey’s complex sample design.

**Results:**

Analyses included 9143 Hispanic children. In total, 24.9% (95% CI: 22.9–27.0%) experienced dental caries, and there were significant associations between household acculturation and oral health. In unadjusted analyses, 32.0% (95% CI: 28.9–35.4%) of children in low acculturation households, 20.3% (95% CI: 16.0–25.4%) of children in moderate acculturation households, and 16.9% (95% CI: 14.2–20.0%) of children in high acculturation households experienced dental caries (*p* < 0.001). In adjusted analyses, children in high acculturation households were significantly less likely than those in low acculturation households to experience dental caries (*p* < 0.001; OR = 0.50; 95% CI: 0.35–0.70). The difference between children in moderate and low acculturation households approached but did not reach statistical significance (*p* = 0.057; OR = 0.69; 95% CI: 0.48–1.01).

**Conclusions:**

A dose-response relationship was observed between household acculturation and the oral health of Hispanic children in the US. As acculturation increases, the likelihood of a child experiencing dental caries decreases. These findings suggest that public health and community-based interventions intended to reduce oral health disparities in Hispanic children would likely be most impactful if the acculturation levels of the children’s households are considered during program development.

## Background

Hispanic children are a growing segment of the United States (US) population. The percentage of children in the US who are Hispanic has increased from 17% in 2000 to 25% in 2016 [1], and nearly a third of Hispanics in the US are under age 18 [2]. The oral health of Hispanic children is of concern, as they are more likely than children of other racial and ethnic groups to experience dental caries (i.e., tooth decay) [3]. Dental caries are associated with orofacial pain and decreased oral-health-related quality of life and, when untreated, caries lead to tooth loss and systemic infection [4]. Oral diseases are considered a major public health issue because of their high prevalence and their negative effects on well-being [5]. Over time, oral health problems may lead to or exacerbate other health conditions including endocarditis, cardiovascular disease, premature birth, and low birth weight [6]. Further, children’s self-esteem [7], school attendance, and ability to communicate are negatively affected by poor oral health [8].

As slightly over one-third of Hispanics in the US are immigrants [1], many Hispanic children reside in households experiencing acculturation. Acculturation is a process involving a shift in attitudes, behaviors, and identity that occurs as persons or groups from different cultural backgrounds come into contact with one another, and it is most often examined in terms of immigrants’ adaptation to a new society [9, 10]. As immigrants become acculturated, their health status may improve or worsen depending on the health condition of interest, the new environments to which the immigrants are exposed, and the adoption of the host country’s normative dietary, physical activity, and other health-related behaviors. Accordingly, changes in oral health are associated with acculturation [11]. A sizable body of research on Hispanic adults suggests that acculturation typically improves the oral health outcomes of this population [11]. Higher levels of acculturation in Hispanic adults in the US are generally associated with a decreased likelihood of orofacial pain [12], dental caries [13–15] and periodontal disease [13, 14, 16–18], although findings related to oral health-related quality of life are inconsistent [19, 20].

Conversely, there is limited research on the association between acculturation and oral health outcomes in Hispanic children in the US. A recent review of the literature on acculturation and oral health identified only four studies examining oral health outcomes in Hispanic children [11], and those studies had significant limitations. Three of the four were based on convenience samples [21–23], and none included children of all ages and dentition categories [24] nor were they nationally representative of Hispanic children from across the US [14, 21–23]. Our own investigation of the literature identified no additional studies on oral health outcomes in Hispanic children. The few studies on oral health and acculturation in children suggest that oral health outcomes generally improve as acculturation increases [14, 21–23], but due to the serious limitations of these studies additional research is needed to confirm these findings.

Given the large and growing number of Hispanic children in the US [2], the associations between acculturation and oral health in Hispanic adults [12–14, 16, 17], and the effects of pediatric oral health on quality of life and other aspects of health [6–8], the limited research on oral health outcomes in Hispanic children is a concern. The current study fills this important research gap. The objective of this study was to examine the associations between household acculturation and pediatric oral health in the Hispanic population using a nationally representative sample of children and including children ages 1 to 17 from all dentition categories (i.e., primary, transitional, and permanent [24]). Results of this study can be used by community interventionists and other dental program personnel to develop, implement, and evaluate programs intended to reduce oral health disparities of Hispanic children of all age groups who reside in households with varying levels of acculturation.

## Methods

This project was reviewed and approved as expedited category research by the Institutional Review Board of the Office of Research Compliance at the University of North Texas Health Science Center.

### Data Source & Analytic Sample

We analyzed data from the 2011–2012 National Survey of Children’s Health (NSCH), a survey sponsored by the Maternal and Child Health Bureau of the US Health Resources and Services Administration. NSCH examines the physical and emotional well-being of children residing in the US who are between the ages of 0 and 17, and NSCH data are nationally representative of non-institutionalized children when weighted and adjusted for the complex sample design. NSCH data contain health-related information about one randomly selected child per sampled household. These data were collected using telephonic interviews of a parent or guardian who was knowledgeable about the healthcare use and health status of the selected child. The 2011–2012 NSCH data included 95,677 child-level interviews [25].

Our analyses included Hispanic children ages 1 to 17; NSCH does not evaluate the presence of dental caries in children younger than 1 year. Additionally, children with missing data on the variables described below were excluded from the analysis.

### Measures

#### Outcome variable

The outcome of interest was oral health as measured by the presence of dental caries, as ascertained with the question “During the past 12 months did [child’s name] have a toothache, decayed teeth, or unfilled cavities?” We considered a response of “Yes” to be indicative of dental caries. We selected this variable as the outcome variable because oral health status in pediatrics is largely surveyed via dental caries indices and protocols [26].

#### Primary explanatory variable

The primary explanatory variable was household acculturation. As NSCH did not directly assess acculturation, we generated a proxy variable by combining information from three other variables: primary language in the household, language in which the interview was conducted, and generational status of the household. Our acculturation variable categorized children into low, moderate, and high household acculturation groups. The low acculturation group consisted of children who resided in a household in which the primary language was not English or the NSCH interview was conducted in a language other than English and the child had at least one foreign-born parent (i.e., first- and second-generation households). The high acculturation group consisted of children who resided in a household in which the primary language was English, the NSCH interview was conducted in English, and the children’s parents were US-born (i.e., third- or higher generation households). The moderate acculturation group consisted of those not falling into either of the prior two categories. Thus, this group consisted of children in households in which a response to a language-related variable suggested that English was not the preferred language and their parents were born in the US, and it also included children for whom responses to both language-related variables suggested a household preference for English and the child had at least one foreign-born parent.

Our measure of acculturation aligns with past research indicating that, in Hispanic youth, there is a marked shift in socialization and acculturation between the second and third generations [27]. Additionally, while we identified no previous study that used an identically constructed variable to assess household acculturation, our approach is consistent with that of several previous studies that also examined the association between oral health and acculturation. Like our method, these studies utilized a combination of variables to capture the extent of acculturation in minority and immigrant populations [11, 28, 29], including household primary language, generational status, nativity status, age at immigration, length of stay, country of birth, and ethnic identification [11, 28]. Our combined measure of language and generational status serves as a reasonable proxy measure of acculturation given that there are strong associations between generational status, language, and acculturation [30]; in fact, generational status and language are included as components of a variety of acculturation measurement tools designed for the Hispanic population [31].

#### Explanatory covariates

Additional variables were included in our statistical model to adjust for potential confounders in the relationship between dental caries and household acculturation. These covariates consisted of demographic and social determinants of pediatric oral health as identified by da Fonseca and Avenetti [32]. Specifically, we included age (categorized based on dentition status [24]), sex, race, federal poverty level, parental/guardian educational status, presence of health insurance, neighborhood maintenance, household structure (i.e., two parents, one parent and one stepparent, single mother, other), number of children in household, neighbors’ helpfulness, special healthcare needs of the child, parental/guardian health status, and whether the child visited a dentist in the previous 12 months.

### Statistical analyses

First, we determined the number of people in each category of the explanatory variables and conducted Pearson’s chi-square tests to examine the unadjusted relationships between the explanatory variables and the likelihood of a child having dental caries. Next, we explored the adjusted association between household acculturation and childhood dental caries using a multiple logistic regression model [33]. We then used the model to estimate the adjusted probability of having dental caries at each level of household acculturation. We calculated these probabilities based on the average predicted probability of having dental caries conditional on all observations having a given value of the household acculturation variable, and we expressed the results as percentages.

We conducted all analyses in Stata ME version 14.1 (College Station, Texas) and assessed significance at the *p* < 0.05 level. All analyses accounted for the complex survey design of the NSCH.

## Results

A total of 9143 children met the inclusion criteria for the study. After weighting and adjusting for complex sample design, these children represented 11,728,637 Hispanic children from across the US. In total, 24.9% (95% CI: 22.9–27.0%) of the children experienced dental caries. These children were categorized into household acculturation levels based on language and generational status based on the logic described previously; information on the observed counts and weighted proportion of children at each level of the language and generational status variables are provided in Table 1. Low levels of acculturation were observed in 49.3% (95% CI: 46.9–51.6%) of Hispanic children. Children in moderate and high acculturation households represented 16.4% (95% CI: 14.8–18.1%) and 34.4% (95% CI: 32.2–36.6%) of the children, respectively.

**Table 1:** Cross-tabulation of the generational status of households and language use, based on data from the National Survey of Children’s Health, 2011–2012, for Hispanic children in the US. The generational status and language variables were used to categorize children into low, moderate, and high household acculturation levels as shown below. The estimated percentages and estimated Ns account for the weighting and complex survey design of the NSCH and are thus nationally representative, while the observations do not account for weighting or complex survey design

Language	Measure	Generational Status of Household			Totals
		1st Generation	2nd Generation	3rd or Higher Generation	
English was not the primary language in the household or the survey was taken in a language other than English	Acculturation Categorization	Low	Low	Moderate	-
	Estimated % of Population	7.9%	41.4%	2.5%	51.8%
	Estimated N in Population	927,178	4,852,472	293,391	6,073,041
	Observations in Sample	563	2847	165	3575
English was the primary language in the household and the survey was taken in English	Acculturation Categorization	Moderate	Moderate	High	-
	Estimated % of Population	0.2%	13.6%	34.4%	48.2%
	Estimated N in Population	27,727	1,598,506	4,029,363	5,655,596
	Observations in Sample	25	1453	4090	5568
Totals	Acculturation Categorization	-	-	-	-
	Estimated % of Population	8.1%	55.0%	36.9%	100.0%
	Estimated N in Population	954,905	6,450,978	4,322,754	11,728,637
	Observations in Sample	588	4300	4255	9143

### Dental caries and acculturation

There were significant associations between household acculturation and dental caries in both unadjusted and adjusted analyses. Table 2 contains the unadjusted bivariate analyses examining the association between dental caries and other variables of interest, including confidence intervals. In unadjusted analyses, the likelihood of dental caries decreased as household acculturation increased. Specifically, 32.0% of children in low acculturation households experienced dental caries, 20.3% of children in moderate acculturation households experienced dental caries, and 16.9% of children in high acculturation households experienced dental caries (*p* < 0.001).

**Table 2:** Unadjusted associations between dental caries and characteristics of Hispanic children residing in the United States (unweighted *n* = 9143)

	Total Sample (Column %)	Dental Caries in Past Year (Row %)		*p*-value
		Had Dental Caries	No Dental Caries	
Unweighted N	*n* = 9143	*n* = 2019	*n* = 7124	
Total Sample of Hispanic Children	100	24.9 1 (22.9, 27.0)	75.10 (73.0, 77.1)	–
Acculturation Group				
Low Acculturation	49.28 (46.9, 51.6)	32.03 (28.9, 35.4)	67.97 (64.6, 71.1)	< 0.001
Moderate Acculturation	16.37 (14.8, 18.1)	20.31 (16.0, 25.4)	79.69 (74.6, 84.0)	
High Acculturation	34.35 (32.2, 36.6)	16.88 (14.2, 20.0)	83.12 (80.0, 85.8)	
Age Range: Dentition Status				
1–5: Deciduous Dentition	30.65 (28.6, 32.8)	14.32 (11.6, 17.6)	85.68 (82.4, 88.4)	< 0.001
6–12: Transitional Dentition	44.22 (41.9, 46.6)	32.38 (29.1, 35.8)	67.62 (64.2, 70.9)	
13–17: Permanent Dentition	25.12 (23.1, 27.2)	24.67 (20.8, 29.0)	75.32 (71.0, 79.2)	
Sex				
Male	51.6 (49.3, 54.0)	23.98 (21.2, 27.0)	76.02 (73.0, 78.8)	0.361
Female	48.4 (46.0, 50.7)	25.90 (23.1, 29.0)	74.10 (71.0, 76.9)	
Race				
White	58.75 (56.4, 61.1)	23.93 (21.4, 26.6)	76.07 (73.4, 78.6)	0.275
Black	3.81 (3.0, 4.8)	20.78 (13.2, 31.2)	79.22 (68.8, 86.9)	
Other	37.43 (35.1, 39.8)	26.87 (23.4, 30.6)	73.13 (69.4, 76.5)	
Federal Poverty Level (FPL)				
<=100% FPL	35.47 (32.3, 37.7)	29.11 (25.8, 32.7)	70.89 (67.3, 74.2)	< 0.001
> 100–150% FPL	16.27 (14.6, 18.1)	32.30 (26.9, 38.2)	67.71 (61.8, 73.1)	
> 150–200% FPL	12.26 (10.7,14.0)	28.91 (22.8, 36.0)	71.09 (64.0, 77.2)	
> 200–400% FPL	21.82 (19.9, 24.0)	19.71 (15.7, 24.4)	80.28 (75.6, 84.3)	
> 400% FPL	14.17 (12.7, 15.7)	10.44 (8.0, 13.5)	89.56 (86.5, 92.0)	
Parent/Guardian Education Status				
Not High School Graduate	50.54 (48.2, 52.9)	30.29 (27.1, 33.7)	69.71 (66.3, 72.9)	< 0.001
High School Graduate	23.36 (21.5, 25.4)	19.58 (27.1, 33.7)	80.41 (76.6, 83.7)	
Education Beyond High School	26.09 (24.2, 28.1)	19.26 (16.3, 23.4)	80.74 (77.3, 83.8)	
Neighborhood Maintenance				
No Dilapidated Housing	82.23 (80.4, 83.9)	23.78 (21.6, 26.1)	76.22 (73.9, 78.4)	0.018
Has Dilapidated Housing	17.77 (16.1, 19.6)	30.14 (25.4, 35.4)	69.86 (64.3, 74.6)	
Health Insurance				
No Insurance	9.87 (8.5, 11.4)	27.43 (21.0, 34.9)	72.57 (65.1, 79.0)	0.044
Insurance	9.01 (88.6, 91.5)	24.63 (22.5, 26.9)	75.68 (73.1, 77.5)	
Past Year Dental Visit				
No Dental Visit	24.15 (22.2, 26.2)	10.40 (8.0, 13.4)	89.60 (85.7, 92.0)	<.0001
Had Dental Visit	75.85 (73.8, 77.8)	29.53 (27.1, 32.1)	70.47 (67.9, 72.9)	
Household Structure				
2 Biological or Adoptive Parents	66.51 (64.3, 68.7)	23.49 (21.1, 26.0)	76.51 (74.0, 78.9)	0.159
2 Parents and 1 is a Stepparent	9.51 (8.2, 11.0)	29.38 (22.6, 37.3)	70.62 (62.7, 77.4)	
Single Mother	20.44 (18.7, 22.3)	28.46 (24.1, 33.2)	71.54 (66.8, 75.9)	
Other	3.55 (2.7, 4.7)	19.10 (9.4, 35.0)	80.91 (65.0, 90.6)	
Number Children in Household (HH)				
1 Child in HH	19.50 (17.9, 21.2)	21.25 (17.5, 25.6)	78.75 (74.2, 82.5)	0.067
> 1 Child in HH	80.50 (78.8, 82.1)	25.79 (23.5, 28.2)	74.21 (71.8, 76.5)	
Neighbors Help Each Other				
Strongly Agree	34.29 (32.1, 36.5)	24.30 (21.0, 27.9)	75.70 (72.1, 79.0)	0.3482
Somewhat Agree	44.67 (42.4, 47.0)	23.98 (21.1, 27.2)	76.02 (72.8, 78.9)	
Disagree	21.03 (19.2, 23.0)	27.87 (23.4, 32.9)	72.13 (67.1, 76.6)	
Child with Special Healthcare Needs (CSHN)				
Not CSHN	83.35 (81.6, 85.0)	24.16 (22.0, 26.5)	75.84 (73.5, 78.0)	0.111
CSHN	16.65 (15.0, 18.4)	28.65 (23.7, 34.2)	71.35 (65.8, 76.2)	
Parent/Guardians‘ Health Status				
All Excellent or Good	74.64 (72.6, 76.6)	21.26 (19.1, 23.6)	78.74 (76.2, 81.9)	< 0.001
≥1 Fair or Poor	25.36 (23.4, 27.4)	35.65 (31.2, 40.3)	64.35 (59.7, 68.8)	

Data are from the 2010–2011 National Survey of Children’s Health (NSHS) and include a sample of Hispanic children ages 1 to 17 residing in the United States. All estimates account for NSCH weighting and complex sampling design and thus are nationally representative. Percentages and 95% confidence intervals given. Unadjusted differences in proportion with dental caries were tested with chi-square tests; *p*-values given

**Fig. 1:**
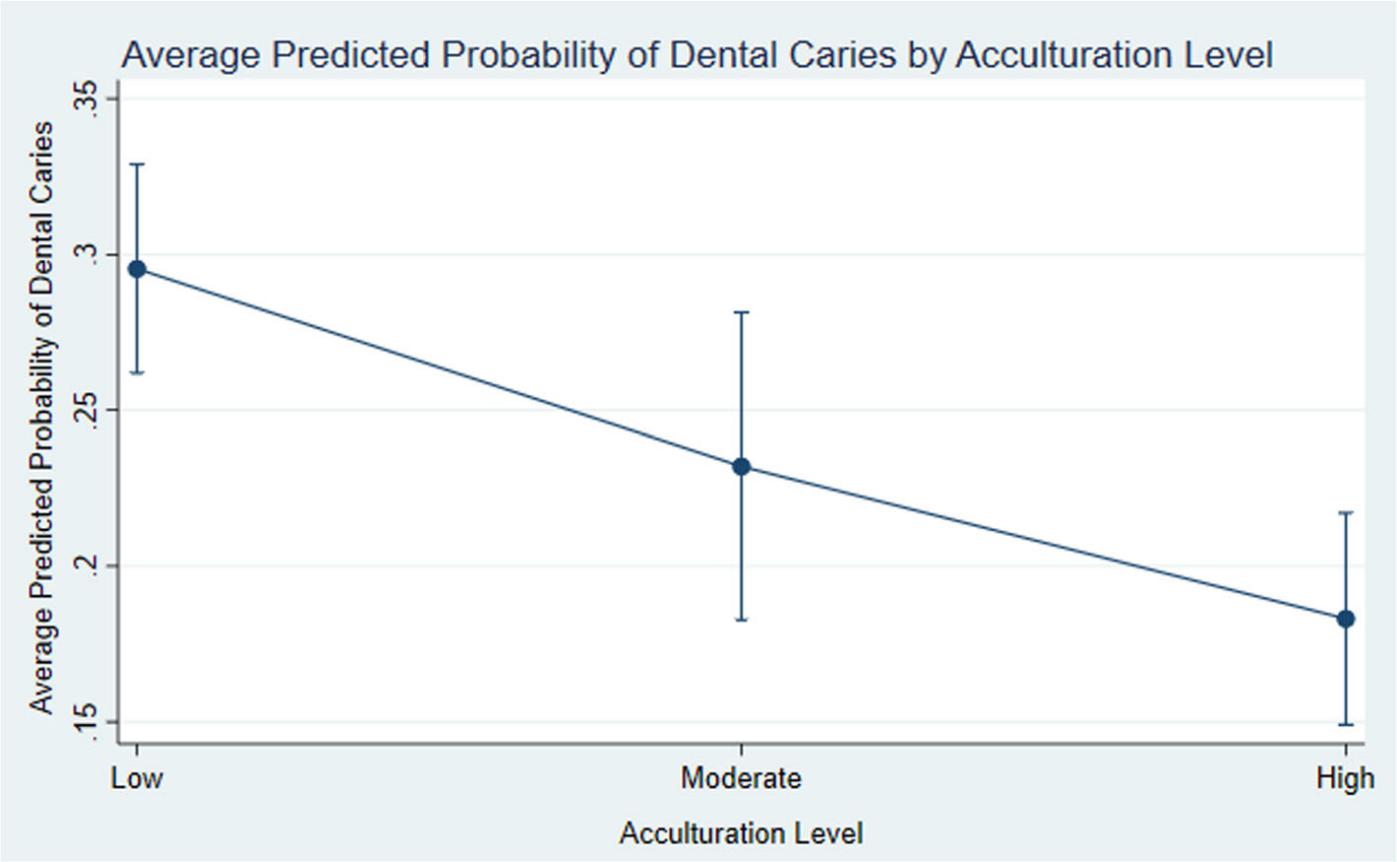
Average predicted probabilities of dental caries by acculturation level in Hispanic children residing in the United States, based on the results of a multivariable logistic regression model that adjusts for demographic and social determinants of pediatric oral health. Detailed model results are available in Table 3

Table 3 contains the results of the logistic regression model examining the adjusted association between household acculturation and childhood dental caries, including odds ratios. In adjusted analyses, a dose-response relationship was observed between acculturation and dental caries (see Fig. 1). Children in high acculturation households were significantly less likely than those in low acculturation households to experience dental caries (*p* < 0.001). The difference between children in moderate and low acculturation households approached but did not reach statistical significance (*p* = 0.057). The average predicted probabilities of dental caries in the high, medium and low acculturation households were 29.5, 23.2, and 18.3%, respectively.

**Table 3:** Results of a logistic regression model examining the adjusted association between dental caries and acculturation in Hispanic children residing in the United States, adjusting for demographic and social determinants of pediatric oral health (unweighted *n* = 9143)

	Odds Ratio (95% CI)	*p*-value	Average Predicted Probability (95% CI)
Household Acculturation			
Low Acculturation	(Ref)		29.5% (26.2, 32.9%)
Moderate Acculturation	0.693 (0.47, 1.01)	0.057	23.2% (18.3, 28.1%)
High Acculturation	0.499 (0.35, 0.70)	0.000	18.3% (14.9, 21.7%)
Age Range: Dentition Status			
1–5: Deciduous Dentition	(Ref)		16.6% (13.2, 19.9%)
6–12: Transitional Dentition	2.39 (1.76, 3.23)	0.000	30.4% (27.4, 33.3%)
13–17: Permanent Dentition	1.65 (1.154, 2.37)	0.006	23.9% (20.1, 27.8%)
Sex			
Male	(Ref)		23.5% (20.9, 26.1%)
Female	1.19 (0.95, 1.50)	0.126	26.4% (23.7, 29.1%)
Race			
White	(Ref)		24.8% (22.4, 27.3%)
Black	0.93 (0.49, 1.76)	0.819	23.6% (13.7, 33.5%)
Other	1.021 (0.87, .80)	0.868	25.2% (22.0, 28.3%)
Federal Poverty Level (FPL)			
< =100% FPL	(Ref)		25.8% (22.4, 29.1%)
> 100–150% FPL	1.29 (0.92, 1.82)	0.146	30.3% (25.0, 35.7%)
> 150–200% FPL	1.19 (0.81, 1.76)	0.371	28.9% (23.0, 34.8%)
> 200–400% FPL	0.82 (.560, 1.21)	0.317	22.5% (17.8, 27.3%)
> 400% FPL	0.45 (0.29, 0.71)	0.001	14.3% (10.1, 18.5%)
Parent/Guardian Education Status			
Not High School Graduate	(Ref)		25.7% (22.6, 28.8%)
High School Graduate	0.85 (0.61, 1.18)	0.333	23.1% (19.2, 27.0%)
Education Beyond High School	0.93 (0.66, 1.31)	0.678	24.5% (20.4, 28.6%)
Neighborhood Maintenance			
No Dilapidated Housing	(Ref)		23.8% (21.7, 25.9%)
Has Dilapidated Housing	1.42 (1.08, 1.90)	0.014	29.9% (25.3, 34.5%)
Health Insurance			
No Insurance	(Ref)		28.1% (20.8, 35.4%)
Insurance	0.810 (0.52, 1.25)	0.343	24.6% (22.6, 26.5%)
Past Year Dental Visit			
No Dental Visit	(Ref)		11.0% (8.4, 13.7%)
Had Dental Visit	3.64 (2.65, 4.98)	0.000	29.0% (26.6, 31.4%)
Household Structure			
2 Biological or Adoptive Parents	(Ref)		23.2% (20.9, 25.5%)
2 Parents and 1 is a Stepparent	1.37 (0.91, 2.05)	0.133	28.4% (21.8, 35.0%)
Single Mother	1.37 (1.03, 1.83)	0.031	28.5% (24.2, 32.8%)
Other	1.22 (0.55, 2.71)	0.627	26.5% (13.0, 39.9%)
Number Children in Household (HH)			
1 Child in HH	(Ref)		24.0% (19.8, 28.1%)
> 1 Child in HH	1.07 (0.80, 1.44)	0.630	25.1% (23.0, 27.3%)
Neighbors Help Each Other			
Strongly Agree	(Ref)		25.9% (22.6, 29.2%)
Somewhat Agree	0.93 (0.71, 1.21)	0.578	24.7% (21.8, 27.6%)
Disagree	0.89 (0.64,1.22)	0.450	23.9% (20.1, 27.3%)
Child with Special Healthcare Needs (CSHN)			
Not CSHN	(Ref)		24.1% (22.1, 26.2%)
CSHN	1.32 (0.96, 1.9)	0.084	28.7% (23.8, 33.7%)
Parent/Guardian Health Status			
All Excellent or Good	(Ref)		22.5% (20.3, 24.7%)
≥ 1 Fair or Poor	1.65 (1.26, 2.16)	0.000	31.1% (27.0, 35.3%)

Data are from the 2010–2011 National Survey of Children’s Health (NSHS) and include a sample of Hispanic children ages 1 to 17 residing in the United States. All estimates account for NSCH weighting and complex sampling design and thus are nationally representative. The outcome of interest was oral health as measured by the presence of dental caries, and household acculturation was the primary explanatory variable. The average predicted probabilities represent the adjusted probability of having dental caries at each category of the predictor variables. They are based on the average predicted probability of having dental caries conditional on all observations having a given value of the predictor variable, and they are expressed as percentages

### Explanatory covariates

Detailed results of the unadjusted and adjusted analyses examining the associations between dental caries and the explanatory variables can be found in Tables 2 and 3, respectively. Neither sex nor race were significantly associated with dental caries in unadjusted nor adjusted analyses (*p* > 0.05 for all). Household poverty level was associated with dental caries in unadjusted analyses (*p* < 0.001). In adjusted analyses, children residing in households with incomes above 400% of the federal poverty level were significantly less likely to experience dental caries than those residing at or below the federal poverty level (*p* = 0.001).

Lower educational levels of the parents/guardians were significantly associated with an increased likelihood of dental caries in unadjusted analyses (p < 0.001), but these differences were not significant in adjusted analyses (*p* > 0.05 for all). Conversely, a lack of neighborhood maintenance was significantly associated with an increased likelihood of dental caries in both unadjusted (*p* = 0.018) and adjusted analyses (*p* = 0.014). Health insurance was not significantly associated with dental caries in either unadjusted (*p* = 0.440) or adjusted (*p* = 0.343) analyses, although past-year dental visits were associated with an increased likelihood of dental caries in both unadjusted and adjusted analyses (*p* < 0.001 for both).

Household structure was not significantly associated with dental caries in unadjusted analyses (*p* = 0.159), but in adjusted analyses children residing in a household with a single mother were significantly more likely to experience dental caries as compared to those living in a household with two biological or adoptive parents (*p* = 0.031). Neither the number of children in the household nor the helpfulness of neighbors was significantly associated with dental caries in the unadjusted or adjusted analyses (*p* > 0.05 for all). Based on unadjusted and adjusted analyses, children with special healthcare needs were not significantly more likely to experience dental caries (*p* = 0.111 and *p* = 0.084, respectively). However, fair or poor health status of parents/guardians was significantly associated with an increased likelihood of dental caries in both unadjusted and adjusted analyses (*p* < 0.001 for both).

## Discussion

The results of our investigation indicate that household acculturation is a significant predictor for dental caries among Hispanic children in the US. These findings concur with existing evidence which projects a general positive and protective effect of acculturation among immigrant and ethnic minorities on oral health-related quality of life [11, 28]. Our investigation adds to the literature by confirming these findings in a nationally representative sample of Hispanic children from all dentition categories. We also demonstrate the presence of a dose-response relationship between the extent of a Hispanic household’s acculturation to the US and pediatric oral health.

The impact of acculturation on oral health must be considered within the context of other health outcomes. The findings of research examining the association between physical health outcomes and acculturation in immigrant and minority populations are inconsistent. Depending on the migration pattern, these vulnerable populations are exposed to a variety of challenges and changes, including language barriers, sociocultural norms, nutrition, and overall lifestyle options and choices. The evidence is mixed regarding the risks and/or protective impacts of acculturation on various health issues and outcomes in immigrant and minority populations, perhaps because acculturation is not dichotomous, but rather reflects a continuum with various stages of adoption [9, 10, 29, 34, 35]. Further, persons will experience varied health consequences depending on the observed behavior changes and the health outcome being studied. For example, some evidence suggests that unrestricted acceptance of westernized norms, particularly diet, leads to an increased risk for chronic health conditions such as cardiovascular disease and diabetes [11]. Contrastingly, evidence also demonstrates a positive correlation between acculturation and utilization of preventive healthcare services, including but not limited to preventive dental health services [36–39]. However, the utilization of healthcare services does not necessarily equate to improvement in health. Literature on Hispanic populations, in particular, suggests a somewhat protective and paradoxical health benefit when Hispanics are resistant to acculturation. Essentially, the concept of the Hispanic Paradox claims that certain sociocentric protective forces operate, intrinsically, within the Hispanic community, including knowledge, attitudes, beliefs, and behaviors pertaining to health and healthcare [29, 40]. The results from our study contests and cautions against using the term “Hispanic Paradox” universally, as it does not appear to apply in the context of oral health.

Our results also highlight the potential role of broader family and community-level factors in affecting oral health, and further investigation in this area is warranted. A major gap that continues to be understudied is the qualitative, social determinants that influence oral health. Parental education, oral health literacy, access to care, material deprivation, poor dietary and hygiene habits, decreased social support, and employment issues are all identified as key areas within social determinants of pediatric oral health [32]. Furthermore, social network theories suggest that health beliefs, behaviors, and values are largely influenced by the context in which people live [41]. For example, the influence of social support systems, communal connectivity, and neighborhood environments are critical factors in predicting the extent of impact sociocultural forces can have on quality of life. In Hispanic communities, studies suggest a protective effect of specific ethnocentric values, namely reciprocity and social relationships, which appears to dissipate as time residing in US increases [40]. Our results support the significant impact of certain social determinants beyond acculturation, specifically household income, household structure, parental/guardian health status, and dilapidated housing environments, on dental caries among Hispanic children in the US. One possible explanation is that proximate connection with certain sociocentric values and identity play a vital role in preserving health. Therefore, future efforts should focus on the extent to which social determinants of health, particularly family structure and extended social networks, influence oral health-related quality of life among Hispanic households in the US.

Cultural competency for healthcare professionals is paramount when striving to provide quality care to a diverse array of patients, particularly immigrant and minority populations. Based on the results from our investigation, less-acculturated Hispanic households are at the highest risk for oral diseases. Clinical and public health professionals should prioritize oral healthcare interventions on less-acculturated Hispanic households. A variety of obstacles to improving health and acquiring healthcare may already exist for Hispanics who are less-acculturated in the US, such as linguistic and financial barriers, ethnocentric health belief systems, navigating national health reform and insurance policies, and more. For immigrant and minority populations, conducting successful preventive interventions often requires a culturally sensitive interprofessional-based and/or community-based approach [29]. Health literacy must also be considered when developing interventions for Hispanic populations, as prior studies indicate that lower levels of acculturation may be associated with lower levels of health literacy [42, 43]. Future studies are needed to explore how health literacy may moderate or mediate the relationship between household acculturation levels and pediatric oral health.

Communication, or lack thereof, another prominent obstacle in the healthcare industry. Evidence suggests that integrating the use of professional interpretation services, incorporating culturally sensitive motivational patient interviewing techniques, employing multilingual staff, utilizing multilingual forms, and regularly collecting patient feedback can help in improving total quality management in medical and dental offices [29]. Moreover, regular interprofessional training and continuing education exercises in cultural competency and literacy-oriented communication can help improve healthcare professionals’ knowledge, awareness, and attitudes towards cultural sensitivities and variations in health literacy across diverse populations. Broader public health-based interventions should include key stakeholders in the community, such as involvement of community health workers who are more acculturated, but also sensitive to the unique needs of the targeted patient populations [29]. Further effort is needed on building more effective health policies and interventions that improve the cultural competence and communication of healthcare practitioners in the US.

Over the past decade, numerous policies have been implemented which strive to ensure that the healthcare needs of persons at varying levels of health literacy are met [44, 45]. In an increasingly diverse national population, there is a need for similar policies that will enable persons at all acculturation levels to receive timely, high quality healthcare. Such policies could help facilitate regular cultural competency-focused training and continuing education for practicing dental health professionals in an effort to reduce existing communication and knowledge gaps. Furthermore, at the academic level, accrediting bodies could make sure that schools teaching future generations of the dental health workforce integrate diversity education and cultural competency as part of their curriculum. At the community level, public health professionals should incorporate acculturation as an integral component of their program implementation strategies [46], and when such programs are focused on dental health in the Hispanic pediatric population these professionals should consider focusing resources on children who reside in less acculturated households given their higher risk of oral health issues. Social media health campaigns appropriately tailored to persons at different levels of acculturation should also be considered in today’s digital age; these campaigns could stress the importance of dental self-care and regular dental healthcare.

Payers, particularly state Medicaid plans, may also play a role in facilitating culturally competent care. Approximately 56% of Hispanic children are enrolled in Medicaid [47], and all Medicaid plans are required to cover dental care for pediatric enrollees [48]. It is likely that the Hispanic children enrolled in Medicaid reside in households with a diverse range of acculturation and health literacy levels, as over half of US-born Hispanic children have at least one parent born outside of the US [49]. With the Centers for Medicare and Medicaid Services asking states to consider outreaching to families of children on Medicaid in order to provide education on the importance of oral health care for children [50], Medicaid plans may be in a unique position to develop and implement oral health-related interventions that accommodate the needs of a large volume of Hispanic children residing in households with varying acculturation and health literacy levels.

Acculturation is an extraordinarily complex, multidimensional, and multidirectional process that shifts the health beliefs, behaviors, and lifestyles of people transitioning between their home and host countries [29]. Given this complexity, there is no single universally accepted parameter to measure the extent of acculturation. Consistent with much of the past research examining acculturation and oral health [13, 15, 16, 19, 21, 23, 35], the current study used a proxy measure of acculturation based on language and nativity. There is a need for future studies with nationally representative samples of children which use validated instruments when examining the association between pediatric oral health and acculturation.

NSCH data provided an opportunity to examine oral health and acculturation in a sizable sample of nationally representative Hispanic children across a broad range of ages. However, this data source also has limitations. Its cross-sectional nature disallows us from making causal statements or examining changes in oral health as the acculturation process occurs, and the question used to measure oral health focused on “toothache, decayed teeth, or unfilled cavities” rather than the full spectrum of potential pediatric oral health issues. Further, oral health was measured based on parental report rather than clinical examination, so there is the possibility that some children with dental caries were not identified as such [51]. Nonetheless, there is a positive correlation between parental report of pediatric oral health and findings from clinical examinations, and valid and reliable oral health information can be obtained from parents regarding their children when data are collected via questionnaire [51].

Missing data was a challenge of the NSCH data; sociodemographic variables were not available for all children, and children with missing data were excluded from analysis. No data were available on oral health-related behaviors, so such behaviors could not be examined in the current study. We were also unable to identify whether children currently or formerly resided in areas without community water fluoridation, and the presence or absence of such fluoridation may have affected the outcome of interest. Additionally, the NSCH survey asks about children’s health insurance coverage, but it does not ask whether insurance coverage includes dental benefits. As having medical coverage is associated with an increase in dental visits even without dental benefit coverage [52], we adjusted for the presence of health insurance in our analyses. However, we were unable to adjust for the presence or absence of dental insurance benefits.

Finally, the NSCH data source did not contain sufficient information to explore how the relationship between acculturation and oral health may vary within the US’s ethnically diverse Hispanic population. In the US, Hispanics of Mexican, Puerto Rican, Salvadorian, Cuban, Dominican, Guatemalan, and Columbian origins each represent subpopulations of greater than 1 million persons, and numerous smaller Hispanic subgroups are also present [1]. Previous research indicates that oral health status varies by Hispanic subpopulation [53]. Further, each subpopulation has a distinct cultural heritage, and thus acculturation processes may vary by subpopulation. The NSCH did not identify the children’s countries or cultures of origin [54], so the exploration of potentially differing relationship between oral health and acculturation within the different subpopulations of Hispanic persons in the US represents an opportunity for future research that builds on the findings of the current study. That said, while Hispanic immigrants come to the US from a variety of countries, the confluence of a common language, human capital disadvantages, and circumstances within the US result in a “distinctive profile for Hispanics as a whole” [55]. There is a great need for public health research on the Hispanic pediatric population, as Hispanic children represent a growing proportion of the US population [1] and a number of health and healthcare disparities are observed when comparing Hispanic and non-Hispanic children [3, 56–58].

Despite the limitations, this study has substantial strengths and it adds to the literature in important ways. The NSCH provides rich data on multiple, intersecting aspects of children’s lives, including the child’s family and social context. Consequently, we were able to account for these factors when examining the relationship between oral health and acculturation in Hispanic children. Further, our study uses a large, nationally representative sample of Hispanic children of across a broad age range, comprising all dentition stages (primary, mixed, and permanent).

## Conclusions

A considerable body of past research suggests that acculturation typically improves oral health outcomes of Hispanic adults in the US [11], but the association between acculturation and oral health in Hispanic children was previously not well-studied. Given the large number of Hispanic children in the US [1], the oral health disparities experienced by these children [8], and the effects of oral health on quality of life and other aspects of children’s health [6], the current study fills an important gap in the pediatric oral health research literature. Based on a nationally representative sample of children, we observed a dose-response relationship between household acculturation and the oral health of Hispanic children in the US. As the level of acculturation increased, Hispanic children’s oral health increased. Thus, children residing in less acculturated households were at the greatest risk of dental caries. Our findings suggest that public health and community-based interventions intended to reduce oral health disparities in Hispanic children are likely to be most impactful if the acculturation levels of the children’s households are considered during program development.

## Data Availability

The 2011–2012 National Survey of Children’s Health dataset used is this study is publicly available on the Centers for Disease Control and Prevention website, at https://www.cdc.gov/nchs/slaits/nsch.htm.

## Abbreviations

CSHN: Child with special healthcare needs
FPL: Federal poverty level
HH: Household
NSCH: National Survey of Children’s Health
US: United States of America
